# Porcine Bocavirus: Achievements in the Past Five Years

**DOI:** 10.3390/v6124946

**Published:** 2014-12-10

**Authors:** Feng Zhou, Haoting Sun, Yuyan Wang

**Affiliations:** 1 Department of Clinical Medicine, Huashan Hospital, Fudan University, Shanghai 200032, China; E-Mails: 09301010100@fudan.edu.cn (F.Z.); sunh09@fudan.edu.cn (H.S.); 2 Department of Medical Microbiology and Parasitology, School of Basic Medical Sciences, Fudan University, Shanghai 200032, China

**Keywords:** porcine bocavirus, *Parvoviridae*, phylogenetic analysis, detection, epidemiology, pathogenesis

## Abstract

Porcine bocavirus is a recently discovered virus that infects pigs and is classified within the *Bocavirus* genus (family *Parvoviridae*, subfamily *Parvovirinae*). The viral genome constitutes linear single-stranded DNA and has three open reading frames that encode four proteins: *NS1*, *NP1*, *VP1*, and *VP2*. There have been more than seven genotypes discovered to date. These genotypes have been classified into three groups based on *VP1* sequence. Porcine bocavirus is much more prevalent in piglets that are co-infected with other pathogens than in healthy piglets. The virus can be detected using PCR, loop-mediated isothermal amplification, cell cultures, indirect immunofluorescence, and other molecular virology techniques. Porcine bocavirus has been detected in various samples, including stool, serum, lymph nodes, and tonsils. Because this virus was discovered only five years ago, there are still many unanswered questions that require further research. This review summarizes the current state of knowledge and primary research achievements regarding porcine bocavirus.

## 1. Introduction

Bocaviruses have been recognized in veterinary medicine since the early 1960s. Bocaviruses can be detected in humans [1,2,3,4,5,6], cattle [7,8,9], canines [10,11], gorillas [12,13,14], cats [15,16], sea lions [17], and possibly other species. The most recently discovered bocaviruses are human bocavirus (HBoV) and porcine bocavirus (PBoV), which have been classified within the genus *Bocavirus* and family *Parvoviridae* [18].

**Figure 1:**
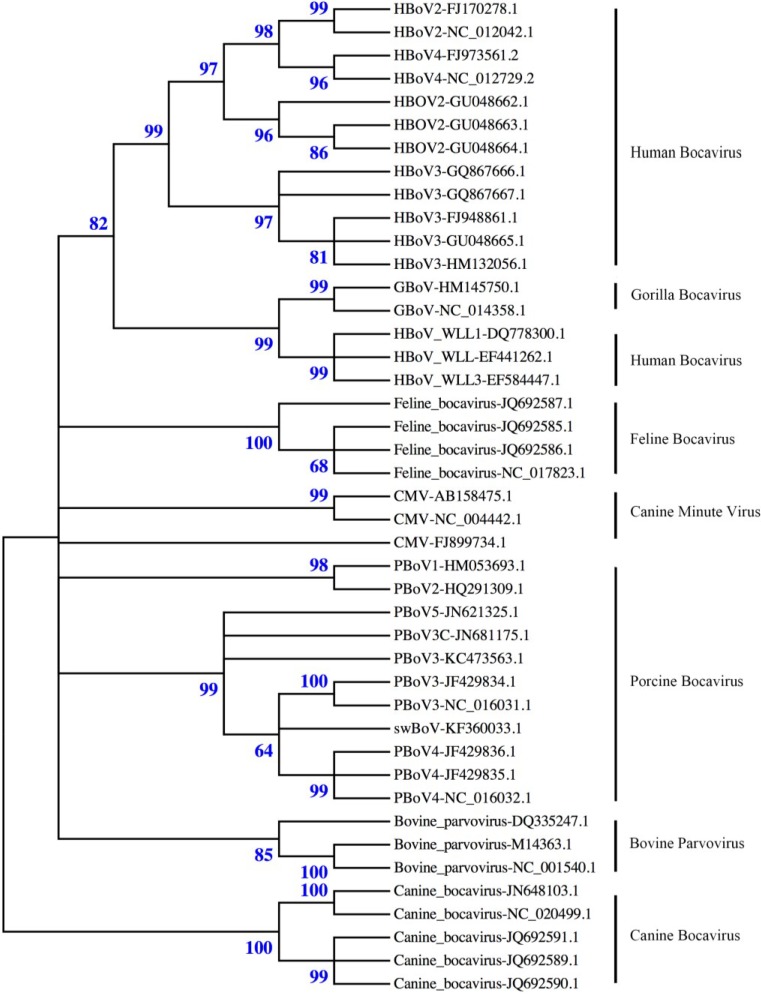
Phylogenetic analysis of bocaviruses. The phylogenetic tree was constructed using nearly full-length nucleotide sequences from bocaviruses in GenBank using the MEGA 5.2 software package [31] (neighbor-joining method with 1000 bootstrap replicates). The relationship among the seven PBoV genotypes (clade Porcine bocavirus) and other bocaviruses can be observed from the tree. PBoV has a close relationship with other bocaviruses, such as feline bocavirus and bovine bocavirus.

In 2009, an 1879-bp sequence of porcine boca-like virus (PBo-likeV) was found in Sweden in lymph nodes from swine with post-weaning multisystemic wasting syndrome (PMWS) [19]. Since this discovery, sequences of bocaviruses have been identified throughout the world. In 2010, the nearly complete genomes of PBoV1/2-CHN were characterized from fecal samples of swine in China, and the partial genomes of two additional boca-like viruses (named 6V and 7V) were also described [20]. In 2011, PBoV3/4-UK was isolated from a primary pig kidney cell culture that was inoculated with a homogenate of small intestine (PBoV3-UK) and the fecal suspension (PBoV4-UK), respectively, of pigs with PMWS from farms in Northern Ireland [21]. Later that year, PBoV3/4-HK was also detected from samples that were obtained in China during the period of 2005-2007 [22]. In 2012, PBoV3C was found with a high detection rate in fecal samples from healthy piglets [23]. In the same year, PBoV5 was discovered in stool from piglets that had clinical diarrhea on a farm in China [24]. In 2013, a novel PBoV strain swBoV CH437 was detected from clinical samples in Northwest China [25]. The phylogenetic relationship between PBoV and the other bocaviruses is shown in Figure 1.

Since its discovery in 2009, PBoV has been detected globally (Table 1). To date, eleven countries, including Sweden, China, the USA, Canada, Mexico, Romania, Hungary, Uganda, Korea, Cameroon, and the UK, have reported infections of PBoV, although the frequency of the reported infections has varied. In the past five years, with developing methods and techniques, the research on PBoV has expanded rapidly. Nevertheless, some questions remain unresolved. This article summarizes research results from the past five years to present a brief review of porcine bocavirus.

## 2. Virus Structure

**Figure 2:**
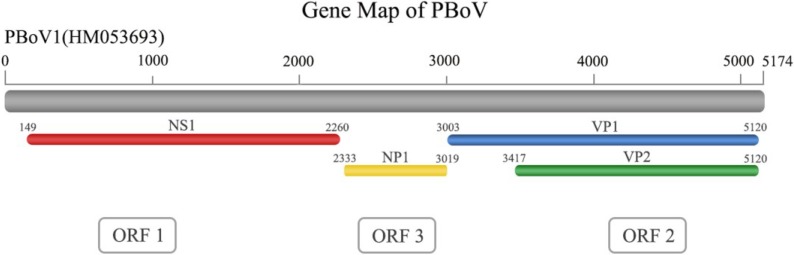
The genome structure of PBoV. The genome of PBoV contains three ORFs that encode four proteins. The sequences for *VP1* and *VP2* overlap in the genome.

Porcine bocaviruses (PBoVs), new members of the *Bocavirus* genus, belong to the family *Parvoviridae* and subfamily *Parvovirinae*. PBoVs are non-lipid enveloped viruses that exhibit icosahedral symmetry and are 25–30 nm in diameter. Their linear single-stranded genome is only ~5 kb in length and contains terminal palindromic sequences [26]. The genome contains three primary open reading frames (ORFs), which are named ORF1, ORF2, and ORF3. ORF1 codes a non-structural protein (*NS1* or replicase) and is located at the 5’-end of the genome. ORF1 has been demonstrated to contain conserved motifs that are associated with rolling-circle replication and helicase and ATPase activities [22]. At the amino acid sequence level, the predicted *NS1* gene of PBoV exhibited sequence identities with the canine minute virus (CMV) of 42.0%–48.5%, bovine parvovirus (BPV) of 30.0%–49.1%, gorilla bocavirus (GBoV) of 40.0%–49.0% and human bocavirus (HBoV) of 38.0%–54.7% [22,27]. *NS1* has been demonstrated to be essential for viral DNA replication in CMV [28,29], which indicates a similar function in PBoV. ORF2 codes the capsid proteins *VP1* and *VP2*, which overlap in the genome (Figure 2). A conserved “YXGXF” motif domain was found in the unique *VP1* protein (*VP1*u) [23]. This domain indicates a secretory phospholipase A_2_ (sPLA_2_) activity that is critical for parvovirus infectivity [20]. Finally, ORF3 codes the NP protein that is located between ORF1 and ORF2 (Figure 2), which is a characteristic genetic feature of the members of the *Bocavirus* genus. Although the function of *NP1* in PBoV has not yet been determined, *NP1* is essential for CMV DNA replication [28]. A recent study has demonstrated that in HBoV, *NP1* blocks IFN production, which suggests a potential immune-evasion mechanism [30].

**Table 1:** Major studies that have reported porcine bocavirus discoveries.

Published Year	First Author	Country	Name	Accession No.
2009	Blomstrom, A.L. [19]	Sweden	PBo-likeV	FJ872544
2010	Cheng, W.X. [20]	China	PBoV1-CHN	HM053693
			PBoV2-CHN	HM053694
			6V	HM053672
			7V	HM053673
2011	McKillen, J. [21]	Northern Ireland	PBoV3-UK	JF512472
			PBoV4-UK	JF512473
2011	Lau, S.K. [22]	China	PBoV3-HK	JF429834
			PBoV4-HK	JF429835
2012	Li, B. [24]	China	PBoV5	JN831651
2012	Yang, W.Z. [23]	China	PBoV3C	JN681175
2014	Wang, E. [25]	China	swBoV CH437	KF360033

In addition to the linear structure of its DNA, PBoV was the second bocavirus in which episomes were detected. The replication of the episome is quite different from that in other parvoviruses [32]. As parvoviruses replicate via a so-called “rolling-hairpin” mechanism that is supported by short imperfectly palindromic hairpin telomeres, their replication intermediates are concatemers of head-to-head or tail-to-tail structures [33,34,35,36]. PBoV and HBoV both replicate in a rolling circle with a head-to-tail structure [32,37]. Despite the different position of hairpin-1 and the miss of hairpin-2 in PBoV, the sequence of the typical structure hairpin-1 is conserved in PBoV. This conserve structure may be related to the identification of replication proteins and binding sites [32].

## 3. Taxonomy and Nomenclature

PBoVs were defined as new species using the ICTV criteria [27], which states that there must be less than 95% homology in the nonstructural gene to be considered a new species in the *Bocavirus* genus. The newly discovered PBoV strains were named in chronological order. As an increasing number of novel PBoV strains are detected, a well-established classification system with specific criteria is essential for further investigation.

Originally, PBoVs were classified into three phylogenetic clades using phylogenetic analysis based on both nucleotide and amino acid alignments [38]. Further studies proposed classification methods based on ORF2-encoded *VP1*, which is a capsid protein and is likely to influence tissue tropism and possibly pathogenesis [39,40]. This method was originally proposed to characterize human bocaviruses using the criteria of >8% amino acid and >10% nucleotide differences from the *VP1* sequences to identify different species [6]. Based on this method, the PBoVs were grouped into four species [39]. In 2012, another classification method based on the *VP1* gene was proposed; in this scheme, PBoV strains with a >40% nucleotide difference from the sequence of the complete *VP1* gene are considered members of different groups, and those with a >10% difference from the *VP1* nucleotide sequence are considered to be members of different subgroups [23]. The subgroups have been defined based on phylogenetic clustering and the homology matrix of known porcine bocaviruses. Using this method, PBoVs can be divided into three groups: PBoV G1, which includes PBo-likeV (FJ872544), PBoV-SX (HQ223038) and PBoV1-H18 (HQ291308); PBoV G2, which includes PBoV1/2-CHN (HM053693/HM053694) and PBoV2-A6 (HQ291309); and PBoV G3, which includes PBoV3/4-UK (JF512472/JF512473), PBoV3/4-HK (JF429834/JF429835), and PBoV3C (JN681175). Group 3 can be further divided into five subgroups: PBoV3A, PBoV3B, PBoV3C, PBoV3D, and PBoV3E, which were previously known as PBoV3-UK, PBoV4-UK, PBoV3C, PBoV3-HK and PBoV4-HK, respectively [23]. In 2013, Xiao *et al.* proposed adding PBoV5 (JN831651) and PBoV3-22/23 (JF713714/JF713715) to PBoV G3. Moreover, different subgroups were proposed; in this classification scheme, PBoV3-UK, PBoV3-22, PBoV4-UK, PBoV3C and PBoV5 each represented a subgroup [41]. Although the five subgroups may need to be re-designated in further studies, this classification, which is mainly based on the *VP1* gene, is widely accepted.

Recently, Huang *et al.* proposed classification methods that are primarily based on the *NS1* gene [42,43]. However, there are differences between this nomenclature and the former nomenclature based on *VP1*. These differences may be due to crossover recombination during the speciation of these viruses [22] and incongruent clustering based on *NS1*, *VP1* and *NP1*.

Given the diversity of known PBoVs, it is very likely that there are more than three groups. More effort should be focused on identifying the best taxonomy and nomenclature.

## 4. Recombination

Different viral strains in the *Parvovirinae* subfamily have been demonstrated to undergo recombination. Phylogenetic analysis has confirmed that a recombination event occurred between HBoV1 and HBoV4, leading to the recombinant strain HBoV2 [44]. Recombination can occur in different ORFs [4,6]. In PBoVs, recombination has also been documented in some regions and strains. Lau *et al.* documented a recombination event between PBoV4-HK and SH14F/12, which may have emerged from recombination between the strains SH14F/1 and SH14F/11 in the *VP1*/*VP2* region [22]. Csagola *et al.* found possible recombination sites at the beginning of the nucleotide sequence between the PBoV1-CHN and PBoV2-CHN genomes. A segment of the genome from nt 227 to nt 278 was replaced in the PBoV2 genomes by PBoV1-CHN sequences (GenBank ID: JN400872, JN400873, JN400874). Moreover, Yang *et al.* have documented recombination in the *NP1* regions of PBoV-WUH1 and PBoV-H18 [23]. Co-infection within species and extensive recombination at the *NP1* and *VP1* gene between and within species have been noted [6]. Taken together, these studies have confirmed that PBoVs undergo recombination, which may lead to changes in the biological characteristics of the virus. More importantly, the possibility of recombination between PBoV and HBoV should be considered.

## 5. Detection of Porcine Bocaviruses Using Molecular Virology Techniques

PBoV can be detected in multiple types of tissue, including lymph nodes [19], serum [28], intestine [21], lung, saliva [45], and spleen [42]. However, given the easy accessibility and generally high positive rate, nasopharyngeal samples (NPS) and fecal samples are typically used for clinical tests to detect the virus [22].

### 5.1. Cell Culture

In 2010, McKillen *et al.* successfully propagated PBoV3/4-UK in primary pig kidney cells. The virus isolates were identified in culture using electron microscopy, immunofluorescence assays, and PCR [21]. However, PBoV replication *in vitro* is not supported by porcine kidney cells (PK-15), swine testicular cells, porcine alveolar macrophages, monkey kidney cells (MARC-145), or human embryonic kidney epithelial cells (HEK293T) [28].

### 5.2. Sequence Detection Assays

Sequence-based assays, including PCR, RT-PCR, loop-mediated isothermal amplification, and high-throughput DNA sequencing, have been established to detect PBoVs. High-sensitivity assays can detect as little as 3.22 fg/µL of viral DNA through PCR [46]. The *VP1* and *VP2* genes are the preferred sequences for primer design. However, PCR-based assays have been qualitative rather than quantitative. Li *et al.* successfully developed a real-time PCR assay that targets the *NP1* gene of PBo-likeV [47]. The assay can provide sensitive (approximately 20 copies) and specific quantification of PBo-likeV in clinical specimens. Semi-quantitative PCR has also been used to identify PBoV episomes [32].

High-throughput sequencing can detect viruses from a large number of samples. Yang *et al.* used this method to successfully identify PBoV3C from piglet fecal samples [23]. The 5235-nucleotide genome can be sequenced using the genome-walking method. Loop-mediated isothermal amplification is another option for detecting PBoV nucleotides. This method is more easily implemented and sensitive compared with conventional PCR assays. The positive and negative samples can be easily distinguished by adding diluted SYBR Green I to the reaction mix, and the results can be observed visually [48].

### 5.3. Indirect Immunofluorescence Assay

Indirect immunofluorescence assays were developed by McKillen *et al.* for *in vitro* use to monitor viral growth in pig kidney cells [21]. Later, the same group developed monoclonal antibodies (mAbs) against two swine bocaviruses (PBoV3/4-UK) that were isolated in cell cultures. Moreover, monoclonal antibodies they developed could be successfully used in antigen-detecting ELISAs that highlight those fractions containing infectious virus within sucrose gradients [49].

## 6. Epidemiology

PBoV infections have been reported in China, Korea, the USA, Mexico, Canada, Northern Ireland, Romania, Hungary, Cameroon, and Uganda (Table 2). Many of the known PBoV genotypes have been reported in China and the USA, including PBoV G1 (PBo-likeV, PBoV-SX, and PBoV1-H18), PBoV G2 (PBoV1/2-CHN and PBoV2-A6), PBoV G3 (PBoV3C, PBoV3/4-HK and PBoV5), and unclassified strains (6V/7V, and swBoV CH437). A recent study demonstrated that positive rates of PBoV (42.0%, or 76/181) in American samples were significantly greater than those (11.4%, or 46/403) in Chinese samples. The geographical distribution of PBoV mainly lies in the eastern and southern coastal areas of China and the central states of the USA. In China and the USA, Jiangsu Province and the state of Minnesota, respectively, were the centers of high PBoV occurrence frequency [50]. However, epidemiological studies outside of China and the USA have been limited. PBoV G1 detections were reported in Sweden, Northern Ireland, Romania, Hungary, Cameroon, and Uganda (Table 2). Northern Ireland is the only country other than the USA and China in which the prevalence of PBoVG3 is high. A discovery of PPV4 [51], which was once considered to be a member of PBoV, was reported in the USA. However, this virus is now considered to belong to a separate clade that is different from the genera *Parvovirus* and *Bocavirus* [41].

The prevalence of PBoV infection also varies with age and season. In terms of age, Zhai *et al.* found that PBo-likeV infection was significantly more prevalent in weaned piglets (69.7%, or 69/99) than in piglets that were not yet weaned (13.6%, or 3/22), adult boars (0%, or 0/20), adult sows (7.7%, or 2/26), and aborted fetuses (0%, or 0/14) (*p* < 0.01, χ^2^ test) [52]. Similarly, in a wild boar population, Cadar *et al.* reported that piglets that were 6–12 months old (77.06%) were three times more likely to be infected with PBo-likeV than piglets that were 12–36 months old (22.94%) (*p* < 0.01) [53]. The low prevalence of PBo-likeV infection in piglets that are less than 6 months of age suggests a passage of protective maternal immunity. The morbidity (50%–100%) and mortality (20%–60%) rates were also higher in piglets that were 15–70 days old than in sows (>1 year old) and boars (>2 years old), for which little to no mortality was observed [52]. The seasonal infection rates of PBoV are higher from March through May (72.7%, or 16/22) than in June through August (28.9%, of 26/90), September through November (38.7%, or 12/31), and December through February (41.7%, or 20/48) (*p* < 0.05, χ^2^ test). However, there was no significant difference in the rates of PBoV infections throughout the summer, autumn, and winter (*p* < 0.05, χ^2^ test) [52].

The contribution of co-morbidities to PBoV infection rates is still not clear. Early evidence suggested that piglets with PMWS may be more likely to develop PBoV infections. Blomstrom *et al.* reported that the positive rate of PBo-likeV in piglets with and without PMWS was 88% and 46%, respectively [54]. Similarly, Zhai *et al.* reported a higher frequency of PBo-likeV infection in pigs that suffered from respiratory tract symptoms and reproductive failure (38.7%) than in healthy pigs (7.3%) [52]. However, the clinical samples used in this study were from sick pigs from nine provinces throughout China, and the sera from healthy pigs were from a single province. Thus, the difference in the rate of PBo-likeV infection based on co-morbidities observed in this study may not be credible.

**Table 2:** Major epidemiological studies of porcine bocaviruses ^a^.

Country	Age	Health Condition ^b^	n	Rate (%)	*PBoV* Type Tested
Uganda [55]	n/g	n/g	95	2.11%	*PBoV* G1
Cameroon [56]	piglet	healthy	50	46%	overall
USA [57]	mixed	mainly E + R	385	58.7%	overall
USA ^c^ [43]	n/g	E + R	203	43.3%	overall
China [52]	piglet	mainly R + P	191	38.70%	*PBoV* G1
		healthy	41	7.30%	*PBoV* G1
China [20]	piglet	healthy	397	12.59%	*PBoV* G2
China [28]	pig	healthy	120	39.17%	*PBoV* G1
China [38]	mixed	healthy	340	63.20%	*PBoV* G1
			340	64.40%	*PBoV* G2
China [39]	pig	clinically sick	128	30.50%	*PBoV* G1
		healthy	38	23.70%	*PBoV* G1
		clinically sick	128	21.90%	*PBoV* G2
		healthy	38	10.50%	*PBoV* G2
		clinically sick	128	38.30%	*6V/7V*
		healthy	38	44.70%	*6V/7V*
China [22]	n/g	sick + D	213	18.31%	*PBoV* G3
		healthy	90	16.67%	*PBoV* G3
China [47]	piglet	mainly with PMWS + D	180	56.10%	*PBoV* G1
		healthy	78	16.70%	*PBoV* G1
China [23]	piglet	healthy	92	57.61%	*PBoV* G2
			92	19.60%	*PBoV3*C
China [58]	piglet	E	884	31.90%	*PBoV* G1
		healthy	266	26.32%	*PBoV* G1
	pig	E	101	27.72%	*PBoV* G1
		healthy	58	24.14%	*PBoV* G1
China [42]	mixed	E + R + G + D	403	11.41%	overall
Korea [45]	mixed	mixed	920	34.9%	overall
		E + R + G	351	37.8%	overall
		healthy	679	14.9	overall
Sweden [19]	piglet	with PMWS	2	100%	*PBoV* G1
Sweden [54]	piglet	with PMWS	34	88.00%	*PBoV* G1
		healthy	24	46.00%	*PBoV* G1
Northern Ireland [21]	piglet	mainly with PMWS	369	8.70%	*PBoV3*-UK
			369	9.50%	*PBoV4*-UK
Romania [53]	wild boar	n/g	470	9.14%	*PBoV* G1
			372	17.74%	*PBoV* G1
Hungary [59]	n/g	sick + healthy	392	1.50%	*PBoV* G1
			392	4.80%	*PBoV* G2
			392	1.80%	*6V*/*7V*

Abbreviations: n, number of samples; n/g, not given. ^a^ Selection criteria: PubMed search using the key words “bocavirus” and “porcine”. ^b^ For sick pigs, several short forms are used: E for enteric symptoms like diarrhea, inappetence; R for respiratory tract symptoms like cough, dyspnea, panting; P for reproductive failure including abortion/stillbirth for sows and low-quality semen for boars; D for deceased or slaughtered; G for general symptoms such as fever, lethargy, wasting, trembling. ^c^ In this study, fecal samples were collected from the USA, Mexico and Canada.

In contrast, previous work from other groups has reported that the prevalence of PBoVs is higher in healthy pigs/piglets. In 2010, Zeng *et al.* reported a high prevalence (39.17%) of PBo-likeV infection in healthy pigs in Hubei Province, China [28]. Another study reported similarly high rates of infection for PBoV1-H18 (63.2%) and PBoV-A6 (64.4%) in China [38]. However, this study used only fecal samples, which usually have a high positive rate, to detect the virus. Other studies have also confirmed that PBoVs, including PBoV1/2-CHN, PBoV3/4-UK, PBoV3/4-HK and the newly reported PBoV3C, are highly prevalent in healthy pigs (Table 2). Therefore, additional studies are required to determine the pig population that is most susceptible to PBoV infection and the role of co-morbid infections in establishing susceptibility.

## 7. Pathogenesis

The pathogenic mechanism of PBoV infection remains unclear because of the limitations of current studies. In particular, although pigs exhibited clinical symptoms such as trembling, fever, testicular atrophy, abortion or death appear to be more susceptible to PBoV infections (Table 2), the role of co-infections in pathogenesis is still undefined. Commonly reported co-infections include PCV2, PTTV, PRRSV, CSFV, PEDV, PKoV, GARV and TGEV (Table 3) [39,52,58,59]. PBo-likeV co-infections have been documented with numerous pathogens; however, pigs that screen positive for PCV2, PTTV, PEDV, and PKoV have the highest incidence (greater than 70%) of PBo-likeV co-infection. Similarly, in samples that tested positive for PBo-likeV, a relatively high rate (greater than 30%) also tested positive for PCV2, PEDV, PKoV, and GARV (Table 2). Although this result likely reflects the high frequency of these viruses in the population at large, it is possible that these particular viruses may provide a biological benefit to PBoV. Interestingly, PBoV (PBoV3/4-UK) becomes cytopathic after being passaged four times through primary pig kidney cells. Cell lysates that exhibited a cytopathic effect were screened using PCR or RT-PCR and tested negative for PCV1, PCV2, and PPV. These results suggest that even if PBoVs are not directly associated with PMWS or other diseases, they may function as a triggering factor for other infectious agents [19].

The clinical specimens used to detect PBoV include sera, lungs, lymph nodes, tonsils, liver, nasopharyngeal swabs, and fecal samples from healthy and diseased pigs. In terms of virus shedding, Li *et al.* reported that a high viral load (greater than 10^5^ copies·mg^−1^) of PBo-likeV was detected in lung and lymph node samples from diseased pigs using a TaqMan-based real-time PCR assay [47]. A high positive rate of PBoV DNA can also be detected in lung, intestinal, and fecal samples and nasopharyngeal swabs [21,53,58], suggesting that *PBoV* may invade these tissues and that there might be an association with other diseases. Consistent with the possible link between PBoV and other pathogens, Zhang *et al.* reported a higher viral load in diarrhea than in healthy fecal samples (4.6 × 10^5^ *versus* 2.9 × 10^5^ copies/g of stool), but the difference was not significant [58]. So far, there is no sound evidence regarding the pathogenesis of PBoV, thus well-designed investigation are recommended in the future.

**Table 3:** Group PBoV1 co-infection in relation to common diarrhea viruses.

	Group *PBoV1*(+) Samples with Copathogen ^b^	Listed Virus(+) Samples Coinfected with *PBoV* ^c^
PCV2 (%)	3.3–83.8	37.7
PTTV1 (%)	73	n/g
PTTV2 (%)	70.3	n/g
PRRSV (%)	0–67.6	27.3
CSFV (%)	6.9–34.5	20.7
PEDV (%)	72.6	34
PKoV (%)	72.1	32.9
GARV (%)	9.9	41
TGEV (%)	1	66.7

Abbreviations: PCV2, porcine circovirus type 2; PTTV, porcine torque teno virus; PRRSV, porcine reproductive and respiratory syndrome virus; CSFV, classical swine fever virus; PEDV, porcine epidemic diarrhea virus; PKoV, porcine kobuvirus; PBoV, porcine bocavirus; GARV, porcine group A rotavirus; TGEV, transmissible gastroenteritis virus. ^a^ Four studies are included [39,52,58,59]. ^b^ Median number of group PBoV1-positive samples that were found to be coinfected with the listed pathogen. ^c^ Median number of samples that tested positive for the listed viruses and were found to be co-infected with group PBoV1.

Because HBoV and PBoV both belong to the genus *Bocavirus* and family *Parvoviridae*, they share many similarities in terms of their virus characteristics. Thus, it is meaningful to apply experience from HBoV research to PBoV, especially now that there are abundant studies regarding the pathogenicity of HBoV. The high prevalence of HBoV DNA in serum is associated with malignant tumors [60]. Moreover, in tissue samples, human bocavirus DNA was detected in 18.3% (11/60) of lung tissue samples and 20.5% (9/44) of colorectal tumors and may be present in the nuclei of infected cells, which indicates the existence of the postulated σ- or rolling-hairpin replication mechanism [61]. Because of the lack of appropriate *in vitro* or *in vivo* models, it is difficult to detect the tumors in pigs even though the PBoVG2 and PBoVG3 episomes have been identified [32] However, it would be useful to determine the potential host organ of PBoV.

## 8. Conclusions

Since the discovery of PBoV five years ago, our knowledge of the virus has been enriched considerably. The primary achievements are the following: (1) knowledge has been widely shared; (2) detailed epidemic data have been collected; (3) genome sequences have been acquired, and the functions of the open reading frames have been preliminarily interpreted; (4) five genotypes of PBoV have been found, and they were classified into three groups; (5) sequence analysis has indicated the relationship between PBoV and other bocaviruses; (6) several detection methods have been developed; and (7) the virus has been successfully grown *in vitro*. Despite these advances, there are still many questions that require further study, and with rapidly advancing tools, these questions may be resolved in the near future.

## Authors Contributions

Feng Zhou contributed to the original draft of the manuscript. Haoting Sun contributed by collecting the data and creating the figures and tables in the manuscript. Yuyan Wang contributed by revising the manuscript. All authors read and approved the final manuscript.
